# The glucocorticoid receptor and cortisol levels in pediatric septic shock

**DOI:** 10.1186/s13054-018-2177-8

**Published:** 2018-09-29

**Authors:** Matthew N. Alder, Amy M. Opoka, Hector R. Wong

**Affiliations:** Division of Critical Care Medicine, Cincinnati Children’s Hospital Medical Center, Children’s Hospital Research Foundation, 3333 Burnet Avenue, MLC 2005, Cincinnati, OH 45229 USA

**Keywords:** Sepsis, Septic shock, Glucocorticoid receptor, Cortisol

## Abstract

**Background:**

There is controversy around the prescription of adjunct corticosteroids in patients with fluid-refractory septic shock, and studies provide mixed results, showing benefit, no benefit, and harm. Traditional means for evaluating whether a patient receives corticosteroids relied on anecdotal experience or measurement of serum cortisol production following stimulation. We set out to measure both serum cortisol and the intracellular signaling receptor for cortisol, the glucocorticoid receptor (GCR), in this group of patients.

**Methods:**

We enrolled pediatric patients admitted to the pediatric intensive care unit with a diagnosis of systemic inflammatory response syndrome (SIRS), sepsis, or septic shock as well as healthy controls. We measured serum cortisol concentration and GCR expression by flow cytometry in peripheral blood leukocytes on the day of admission and day 3.

**Results:**

We enrolled 164 patients for analysis. There was no difference between GCR expression comparing SIRS, sepsis, and septic shock. When all patients with septic shock were compared, those patients with a complicated course, defined as two or more organ failures at day 7 or death by day 28, had lower expression of GCR in all peripheral blood leukocytes. Further analysis suggested that patients with the combination of low GCR and high serum cortisol had higher rates of complicated course (75%) compared with the other three possible combinations of GCR and cortisol levels: low GCR and low cortisol (33%), high GCR and high cortisol (33%), and high GCR and low cortisol (13%; *P* <0.05).

**Conclusions:**

We show that decreased expression of the GCR correlated with poor outcome from septic shock, particularly in those patients with high serum cortisol. This is consistent with findings from transcriptional studies showing that downregulation of GCR signaling genes portends worse outcome.

**Electronic supplementary material:**

The online version of this article (10.1186/s13054-018-2177-8) contains supplementary material, which is available to authorized users.

## Background

Pediatric septic shock remains a major cause of morbidity and mortality throughout the world [1]. Even with the highest level of care, many children succumb to septic shock despite years of research and millions of dollars spent. Heterogeneity among patients with septic shock has resulted in our inability to clearly show beneficial interventions beyond antibiotics and supportive care. This is nowhere more obvious than in our current practice of prescribing adjunctive corticosteroids for patients with refractory septic shock. In an era of precision medicine, we need tools that allow us to better classify patients with septic shock to allow evidence-based practice rather than anecdotal experience.

Whether or not to prescribe corticosteroids for patients with fluid-refractory septic shock has been questioned for more than four decades, and no definitive answer currently exists. Many reviews have summarized these studies [2–5]. Some studies have suggested benefit [6], but subsequent trials have not been able to confirm these findings [7], whereas other studies suggest that there may be harm due to administration of corticosteroids [8–11]. This was further reiterated with two recent large studies published side by side in the *New England Journal of Medicine*, again showing conflicting results [12, 13]. Heterogeneity within the septic shock population undoubtedly plays into our confusion in trial results, as patients present with different pathophysiology, pathogens, and comorbidities. Until a more definitive trial can better categorize patients into more than just septic shock or no septic shock, we are unlikely to be able to resolve this question.

For some time, our group has focused on the transcriptional characteristics of pediatric patients with septic shock to attempt to de-convolute the heterogeneity of patients [14, 15]. This approach has allowed the subclassification of patients into different risk strata and separate endotypes based on a 100-gene panel [16]. These studies have consistently shown that those patients with downregulation of genes in the glucocorticoid signaling pathway have worse outcomes and that corticosteroid prescription is associated with poor outcome in this group. It is unclear why these patients have downregulation of the glucocorticoid pathway or whether this downregulation directly contributes to poor outcomes.

The primary receptor for the glucocorticoid pathway is the glucocorticoid receptor (GCR), which exists in the cytoplasm of most cells and can influence expression of up to 20% of the genes in peripheral blood cells [3, 17, 18]. The two predominant isoforms of the GCR are GCR alpha and GCR beta. Functionality of the GCR signaling axis comes primarily through the major isoform GCR alpha. The second isoform, GCR beta, is generated by the use of an alternative final exon and creates a dominant negative GCR. We sought to test the relationship between the primary signaling molecule of the glucocorticoid pathway, cortisol, and the primary receptor, GCR alpha, to test whether we could better understand regulation of the glucocorticoid axis in patients with septic shock.

## Methods

### Prospective enrollment of study subjects and data collection

The institutional review board of Cincinnati Children’s Hospital Medical Center approved the protocol for the prospective collection of human blood samples and clinical data. Patients not older than 18 years of age admitted to the pediatric intensive care unit (PICU) and meeting pediatric-specific consensus criteria for systemic inflammatory response syndrome (SIRS), sepsis, or septic shock were eligible for enrollment. There were no exclusion criteria for enrollment. Legal guardians were approached for informed consent prior to data or sample collection. For control samples, children presenting for elective hernia repair were approached for willingness to donate blood at the time of intravenous catheter placement. All control subjects were screened to ensure good health and no febrile illness, corticosteroids, or anti-inflammatory medication in the prior two weeks.

“Day 1” samples were obtained within the first 24 hours of meeting criteria for SIRS, sepsis, or septic shock in the PICU; for most samples, this was at presentation to the PICU. “Day 3” samples were obtained 48 hours later. While patients were in the PICU, clinical and laboratory data were collected. Evidence for organ failure was tracked for up to 7 days by using previously published criteria [19], except for mortality, which was tracked for 28 days after enrollment. Illness severity was estimated by using the Pediatric Risk of Mortality (PRISM) score, which is based on laboratory values and clinical variables at the time of enrollment.

### Flow cytometry

Whole blood samples were stained in accordance with standard protocols used for intracellular staining of cytosolic proteins. Briefly, blood samples underwent red cell lysis using ACK buffer (Thermo Fisher, Waltham, MA, USA) followed by washing. Cells were blocked with 10% human serum in flow buffer—phosphate-buffered saline (PBS) with 1% bovine serum albumin (BSA)—and surface-stained with CD3, CD14, and CD66b (Becton Dickinson, Franklin Lakes, NJ, USA). After surface stain, cells were washed and fixed with 2% paraformaldehyde. Cells were washed and permeabilized with intracellular cytokine staining buffer (ICCS Buffer-10 mM HEPES, 0.1% BSA, 0.1% saponin, 0.1% Azide in PBS). Cells were again blocked with 10% human serum and followed by staining for intracellular GCR alpha (Abcam, Cambridge, UK). Stained cells were analyzed on a Canto II flow cytometer (Becton Dickinson). To ensure comparable mean fluorescence intensity (MFI) for samples collected over a long period of time, each time a sample was run, Rainbow Calibration Particles (Becton Dickinson) were used to establish consistent voltage settings.

### Serum cortisol measurement

Cortisol levels were measured from serum samples in the hospital’s clinical lab by using the Access Cortisol Assay (Beckman Coulter, Brea, CA, USA) on the Access 2 immunoanalyzer.

### Statistical analysis

Statistical procedures used SigmaStat Software (Systat Software, Inc., San Jose, CA, USA). Comparisons between groups used the Mann–Whitney *U* test, rank sum test, chi-squared test, or Fisher’s exact test, as appropriate. The association between GCR alpha expression and serum cortisol concentrations was evaluated by using linear regression and all available day 1 and day 3 paired data. The association between GCR alpha expression and outcome was modeled by using multivariable regression, adjusting for illness severity, exposure to corticosteroids, age, and comorbidity. The primary outcome variable for the regression procedures was a complicated course, defined as the persistence of two or more organ failures at day 7 of septic shock or 28-day mortality. Since this was an exploratory study, *a priori* we planned to extend our initial analysis, guided by the findings. For ease of reference, we describe exploratory analyses in the Results section.

## Results

Table 1 shows the clinical and demographic characteristics of the study cohort. Subjects with sepsis and septic shock were older than controls. Patients with septic shock had higher baseline cortisol concentrations, higher PRISM scores, and a higher rate of a complicated course. No other differences were noted.

**Table 1:** Demographic and clinical variables of the study cohort

	Controls	SIRS	Sepsis	Septic shock
N	35	17	23	89
Median age, years (IQR)	2.0 (0.6–5.0)	5.7 (3.3–10.8)	6.6 (3.8–14.3)^a^	7.4 (1.7–14.2)^a^
Males, *n* (%)	24 (69)	8 (47)	11 (48)	53 (60)
Median baseline cortisol, μg/dL (IQR)	15 (9–21)	27 (14–35)	19 (10–30)	25 (13–49)^a^
Mortality, *n* (%)	–	2 (12)	0 (0)	6 (8)
Complicated course, *n* (%)	–	2 (12)	0 (0)	21 (24)^b^
Median PRISM, (IQR)	–	4 (3–11)	5 (3–8)	11 (7–15)^c^
Gram-positive bacteria, *n* (%)	–	–	10 (43)	15 (17)
Gram-negative bacteria, *n* (%)	–	–	0 (0)	19 (21)
Other organism, *n* (%)^d^	–	–	0 (0)	8 (9)
Culture negative, *n* (%)	–	–	13 (57)	47 (53)
Comorbidity, *n* (%)	–	14 (82)	16 (70)	60 (67)
Malignancy, *n* (%)	–	0 (0)	3 (13)	14 (16)
Immunosuppression, *n* (%)^e^	–	1 (6)	3 (13)	20 (22)
Bone marrow transplantation, *n* (%)	–	0 (0)	1 (4)	13 (15)
Received corticosteroids, *n* (%)	–	2 (12)	7 (30)	37 (42)

^a^*P* <0.05 versus controls, analysis of variance (ANOVA) on ranks

^b^*P* <0.05, chi-squared, 2 degrees of freedom

^c^*P* <0.05 versus systemic inflammatory response syndrome (SIRS) and sepsis, ANOVA on ranks

^d^Refers to viral, fungal, or mixed infections

^e^Refers to patients who were status post bone marrow transplantation, patients with malignancies and bone marrow suppression, and patients receiving immunosuppression medications following solid organ transplantation.

Abbreviations: *IQR* interquartile range, *PRISM* Pediatric Risk of Mortality

Additional file 1 shows the GCR alpha MFI across all four study groups, for all cells combined, as well as subpopulations of leukocytes, lymphocytes, monocytes, neutrophils, on day 1 and day 3 of admission. GCR alpha expression was greater than that of controls for some combinations of cell types and study categories, but no consistent pattern was noted. GCR alpha expression was not different between subjects in the SIRS, sepsis, and septic shock groups.

Table 2 compares GCR alpha expression among the subjects with septic shock, grouped into those with and without a complicated course. No differences were noted on day 1. In contrast, on day 3, GCR alpha expression was significantly decreased among subjects with a complicated course. This was evident for all white blood cells as well as the individual white blood cell populations.

**Table 2:** Glucocorticoid receptor alpha expression table comparing patients with septic shock, with and without a complicated course

Cell type	Day	Non-complicated course	Complicated course
All white blood cells	1	1965 (1080–2780)	1830 (1318–3506)
All white blood cells	3	3019 (1741–3474)	1792 (1224–2538)^a^
Lymphocytes	1	878 (441–1243)	773 (342–1182)
Lymphocytes	3	1014 (798–1619)	714 (304–1267)^a^
Monocytes	1	3035 (1954–3566)	2495 (1601–4525)
Monocytes	3	3669 (3264–4375)	2557 (1564–3388)^a^
Neutrophils	1	2320 (1474–3039)	1981 (1440–3600)
Neutrophils	3	3497 (2420–4062)	2067 (1518–2845)^a^

Shown as median mean fluorescence intensity (interquartile range). ^a^*P* <0.05 versus non-complicated course, rank sum test

Based on the observation that day 3 GCR alpha expression was consistently decreased among patients with septic shock and a complicated course, we next used logistic regression to further assess the association between GCR alpha expression on all white blood cells and a complicated course. As shown in Table 3, by univariable analysis, higher day 3 GCR alpha expression was associated with decreased odds of a complicated course. Higher PRISM score and exposure to exogenous corticosteroids were associated with increased odds of a complicated course. Age and the presence of a comorbidity were not associated with a complicated course. We next conducted a multivariable analysis to determine whether day 3 GCR alpha expression, PRISM score, and corticosteroid exposure were independently associated with a complicated course (Table 3). Although there were trends, none of the three variables was independently associated with a complicated course. This suggests co-linearity and complex interactions between GCR alpha expression, illness severity, and corticosteroid exposure. When we considered interaction terms between the three variables, none of the interaction terms was associated with a complicated course (data not shown).

**Table 3:** Univariable and multivariable logistic regression to test for associations between the listed variables and a complicated course

Variable	Univariable analysis			Multivariable analysis		
	Odds ratio	95% CI	*P* value	Odds ratio	95% CI	*P* value
Day 3 GCR alpha MFI	0.03	0.00 to 0.85	0.039	0.049	0.00 to 2.12	0.117
PRISM	1.3	1.1 to 1.5	0.002	1.2	1.0 to 1.4	0.058
Corticosteroids	4.8	1.4 to 16.2	0.012	3.7	0.8 to 16.8	0.092
Age	1.0	0.9 to 1.1	0.454	–	–	–
Comorbidity	2.0	0.5 to 7.4	0.317	–	–	–
Bone marrow transplantation	1.6	0.6 to 4.3	0.393	–	–	–
Immunosuppression	0.4	0.3 to 2.1	0.640			

Abbreviations: *CI* confidence interval, *GCR* glucocorticoid receptor, *MFI* mean fluorescence intensity, *PRISM* Pediatric Risk of Mortality

**Fig. 1:**
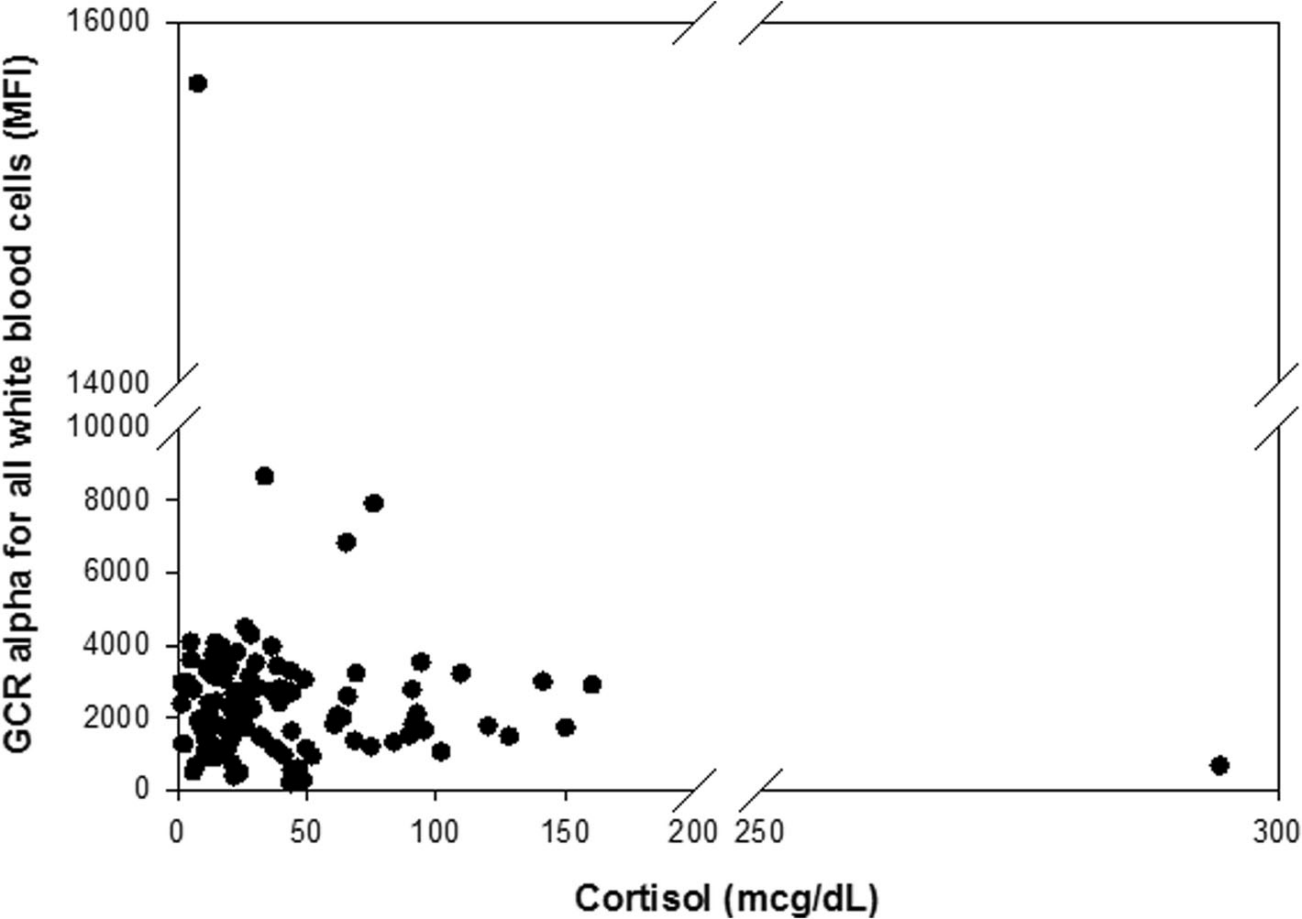
No correlation between glucocorticoid receptor (GCR) alpha and serum cortisol. Comparison of flow cytometric mean fluorescence intensity (MFI) of all leukocyte GCR alpha expression relative to serum cortisol (*R* = 0.058, *P* = 0.532)

We then sought to test whether there was a relationship between GCR alpha expression and serum cortisol concentration among patients with septic shock. When all patients, regardless of whether they received exogenous corticosteroids, were considered by linear regression, there was no association between serum cortisol concentration and GCR expression from all cells (Fig. 1; *R* = 0.058, *P* = 0.532). This lack of association was also evident in the subgroup that received exogenous corticosteroids (*R* = 0.183, *P* = 0.278) and the subgroup that did not receive exogenous corticosteroids (*R* = 0.264, *P* = 0.051). In addition, this lack of association was evident when excluding patients with immunosuppression (*R* = 0.067, *P* = 0.580). Since GCR expression and serum cortisol were not related or had only a weak trend (in the subgroup that did not receive corticosteroids), we next categorized patients into groups of high or low cortisol and high or low GCR expression. To do this, we used the median values of cortisol and GCR MFI for all patients with septic shock. Values above the median were considered “high expression”, and values below the median were considered “low expression”. Table 4 compares indices of illness severity among these four groups. Subjects with low serum cortisol and high GCR expression tended to be less severely ill. In contrast, subjects with high serum cortisol and low GCR expression on day 3 had a significantly higher rate of a complicated course, higher PRISM scores, fewer PICU-free days, and fewer vasopressor-free days. Among the 14 subjects with high serum cortisol and low GCR expression on day 1 who also had available day 3 data, nine remained in the high serum cortisol and low GCR expression group on day 3 and seven of these subjects (78%) had a complicated course. Among the remaining subjects with high serum cortisol and low GCR expression on day 1, but who transitioned to one of the other three possible groups by day 3, none had a complicated course.

**Table 4:** Indices of illness severity among the cortisol high/low and glucocorticoid receptor alpha high/low groups

	High/High	High/Low	Low/High	Low/Low
Day 1 relative cortisol level/relative GCR alpha level				
Number	20	26	13	30
Rate of complicated course	30%	27%	8%	23%
Median PRISM, (IQR)	13 (8–19)	13 (7–18)	9 (4–11)	10 (7–14)
Maximum organ failures, median (IQR)	2 (2–3)	2 (1–3)	2 (1–3)	2 (1–2)
Median PICU-free days, (IQR)	20 (15–26)	23 (9–26)	24 (21–27)	24 (20–26)
Median vasopressor-free days, (IQR)	26 (22–27)	26 (23–27)	27 (25–27)	26 (25–27)
Day 3 relative cortisol level/relative GCR alpha level				
N	12	12	15	12
Rate of complicated course	33%	75%^a^	13%	33%
Median PRISM, (IQR)	13 (10–14)	20 (14–24)^b^	8 (6–12)	10 (8–14)
Maximum organ failures, median (IQR)	2 (2–3)	3 (2–4)^b^	2 (2–2)	2 (2–2)
Median PICU-free days, (IQR)	20 (17–25)	0 (0–13)^c^	21 (10–25)	22 (9–25)
Median vasopressor-free days, (IQR)	25 (23–27)	18 (0–24)^c^	25 (25–27)	25 (24–26)

^a^*P* <0.05, chi-squared, 3 degrees of freedom

^b^*P* <0.05, versus low/high and low/low groups, analysis of variance (ANOVA) on ranks

^c^*P* <0.05, versus all other groups, ANOVA on ranks

Abbreviations: *GCR* glucocorticoid receptor, *IQR* interquartile range, *PICU* pediatric intensive care unit, *PRISM* Pediatric Risk of Mortality

## Discussion

The current recommendation for patients with fluid- and vasopressor-refractory septic shock is to consider treatment with corticosteroids [20, 21]. However, there is a lack of consensus and conclusive evidence that corticosteroids are beneficial to all pediatric patients with septic shock. We postulated that evaluating serum cortisol alone or the response to stimulation with adrenocorticotropic hormone (ACTH) was insufficient to predict which patients would respond to corticosteroids. As a first step toward testing this hypothesis, we characterized a cohort of pediatric patients and tested expression of both cortisol and the intracellular GCR. This hypothesis was based on our previous findings that patients with downregulation of the glucocorticoid signaling genes had worse outcomes.

GCR alpha measurement in this patient cohort did not show any consistent patterns when comparing patients with different diagnoses, SIRS, sepsis, or septic shock. Some white blood cell populations, such as lymphocytes, did show increased GCR expression with any diagnosis at the time of admission, but no other reliable trend was present (Additional file 1). Further subclassification of patients with septic shock into those with a complicated and non-complicated course revealed that those patients who had a complicated course had lower expression of GCR alpha. Admission samples tended to be lower in those patients with a complicated course but this became significant only at day 3 (Table 2). Univariate analysis further confirmed that high expression of GCR alpha decreased the odds of a complicated course (Table 3). This finding is consistent with our previous transcriptional studies showing that patients with a complicated course had downregulation of genes in the glucocorticoid signaling pathway [11, 16]. Other groups have also shown reduced GCR alpha expression in pediatric patients critically ill with septic shock [22].

We found no correlation between serum cortisol level and the GCR alpha receptor. This suggests that these two important molecules are subject to independent regulation during critical illness. For this reason, we further subdivided patients into groups with high and low expression of cortisol and GCR alpha. This method of grouping patients yielded two interesting findings. First, those patients with low cortisol levels and high GCR expression on day 1 or day 3 had the lowest rates of complicated course. This finding seems consistent with patients with an overall low level of stress response to infection demonstrated by low cortisol level and high GCR alpha receptor available for adequate signaling. The group with the exact opposite levels, high cortisol and low GCR expression, had the worst outcomes with much higher rates of complicated course (Table 4) and this association appeared to be strongest among those who persisted with high cortisol and low GCR expression from day 1 to day 3. This finding suggests a subgroup of patients with persistent high levels of physiological and biological stress, as indicated by high production of cortisol, who have concomitant low expression of the GCR and therefore may be unable to adequately respond to the stress signal. If this is true, then it would be difficult to conceive how prescription of corticosteroids could be of benefit for this subgroup.

We choose to focus on GCR alpha because it is the primary signaling molecule of the glucocorticoid axis. We also evaluated GCR beta, but low overall expression and inconsistent intracellular staining in peripheral blood cells prevented meaningful interpretation of the data. In future studies, characterization of GCR beta may further clarify those patients who will not benefit from corticosteroid treatment as those cells with high GCR beta would be expected not to respond to glucocorticoid treatment as GCR beta is a dominant negative receptor [23, 24].

A weakness of this study is that we were able to measure GCR expression only in peripheral blood cells, which may not be the most important cells for determining the physiologic effects of cortisol on and the glucocorticoid signaling axis. Evaluating GCR expression in tissue, particularly vasculature, may be more meaningful, but inaccessibility is a major impediment. Few studies have looked at tissue expression of GCR alpha and beta [25]; however, these studies are also complicated by many cell types, and differing levels of GCR expression make meaningful interpretation difficult. In addition, not all of the patients with septic shock had an available day 3 sample because they were discharged from the PICU and no longer had vascular access for the study blood draws. This raises the possibility of unintended selection bias. Finally, we collected samples as soon as possible after admission to the PICU to attempt to measure levels as close as possible to the diagnosis of septic shock. However, this resulted in samples being collected throughout the day. The potential for variation in cortisol levels on the basis of circadian rhythm is not accounted for in this approach.

## Conclusions

We have shown differential expression of two of the major glucocorticoid signaling molecules—cortisol and GCR alpha—in critically ill children. The finding of low expression of GCR in patients with a complicated course matches previous studies showing that patients with downregulation of this axis have worse outcomes. These studies may account for why the ACTH stimulation test alone does not explain which patients will benefit from corticosteroids as the ability to generate an appropriate level of cortisol during stress also requires a receptor for the cortisol to have an effect. Some patients with low GCR expression already have high serum cortisol, calling into question whether exogenous corticosteroids will be of any benefit to this group of patients. On the other hand, there are patients with high GCR expression but low cortisol who may very well benefit from additional corticosteroids. Future studies will need to look at GCR expression in specific diseases that have more consistently shown benefit from treatment with corticosteroids, such as dengue fever. The ultimate answer may not come until we are able to study these subgroups of patients in a clinical trial. As momentum builds for a randomized pediatric trial testing the role of corticosteroids in septic shock, perhaps evaluating intracellular expression of GCR would be beneficial.

## Additional file

Additional file 1: Glucocorticoid receptor (GCR) alpha expression table for all study groups. Shown as median mean fluorescence intensity (MFI) (interquartile range, or IQR). Contains GCR alpha MFI data for all patients evaluated in the cohort. Includes evaluating all cells together as well as individual cell populations. (DOCX 13 kb)

## Abbreviations

ACTH: Adrenocorticotropic hormone
GCR: Glucocorticoid receptor
MFI: Mean fluorescence intensity
PICU: Pediatric intensive care unit
PRISM: Pediatric risk of mortality
SIRS: Systemic inflammatory response syndrome
