# Supplementary material for: The glucocorticoid receptor and cortisol levels in pediatric septic shock
Source: Crit Care. 2018 Sep 29;22:244. doi: 10.1186/s13054-018-2177-8 (PMC6162875; doi:10.1186/s13054-018-2177-8)
Supplement: Supplementary file 1 — Glucocorticoid receptor (GCR) alpha expression table for all study groups. Shown as median mean fluorescence intensity (MFI) (interquartile range, or IQR). Contains GCR alpha MFI data for all patients evaluated in the cohort. Includes evaluating all cells together as well as individual cell populations. (DOCX 13 kb) [file 13054_2018_2177_MOESM1_ESM.docx]

**Supplemental Table 1:** GCR alpha expression table for all study groups. Shown as median MFI (IQR).

| **Cell Type** | **Day** | **Controls** | **SIRS** | **Sepsis** | **Septic Shock** |
| --- | --- | --- | --- | --- | --- |
| All white blood cells | 1 | 1194 (774 – 1611) | 1456 (971 – 2429) | 2412 (1394 – 2674)^a^ | 1879 (1136 – 2815) |
| All white blood cells | 3 | -- | 1556 (1357 – 1865) | 1627 (771 – 4384) | 2220 (1475 – 3381)^a^ |
|  |  |  |  |  |  |
| Lymphocytes | 1 | 488 (325 – 681) | 874 (633 – 1346)^a^ | 830 (655 – 1318)^a^ | 873 (440 – 1224)^a^ |
| Lymphocytes | 3 | -- | 840 (695 – 965) | 803 (153 – 1980) | 890 (554 – 1556)^a^ |
|  |  |  |  |  |  |
| Monocytes | 1 | 2773 (1978 – 3957) | 2487 (2243 – 3479) | 3023 (2174 – 4329) | 2967 (1881 – 3943) |
| Monocytes | 3 | -- | 2891 (1978 – 3957) | 3046 (1319 – 5665) | 3440 (2447 – 4267) |
|  |  |  |  |  |  |
| Neutrophils | 1 | 1850 (1279 – 2801) | 1732 (1053 – 2995) | 2644 (1656 – 2186) | 2060 (1472 – 3062) |
| Neutrophils | 3 | -- | 1850 (1279 – 2801) | 1843 (1332 – 2207) | 2896 (1832 – 3889)^a^ |

^a^p < 0.05 vs. controls, ANOVA on Ranks
